# Fracture toughness of porous materials – Experimental methods and data

**DOI:** 10.1016/j.dib.2019.103709

**Published:** 2019-03-07

**Authors:** Hans Jelitto, Gerold A. Schneider

**Affiliations:** Hamburg University of Technology, Institute of Advanced Ceramics, Denickestrasse 15, 21073, Hamburg, Germany

**Keywords:** Fracture toughness, Toughness, Elastic properties, Porosity, Modeling

## Abstract

We provide numerical experimental data and detailed information about the sample preparation and the experimental methods, used by different research groups for measuring the fracture toughness of porous materials. These data are supplemental information to the publication “A Geometric Model for the Fracture Toughness of Porous Materials,” [1], which is based on experimental data of ceramic and polymer materials. For the sake of completeness, we provide here also data from fracturing metallic foams. The corresponding theoretical curves, which are based on the model described in the reference, are given additionally in the diagrams. The utilized publications are not a comprehensive compilation of all corresponding measurements concerning porous materials, but should be seen as a typical set of respective experiments with the focus on the fracture toughness of porous materials. The discussion and interpretation are provided in the above-mentioned reference.

## Graphical abstract

**Figure:**
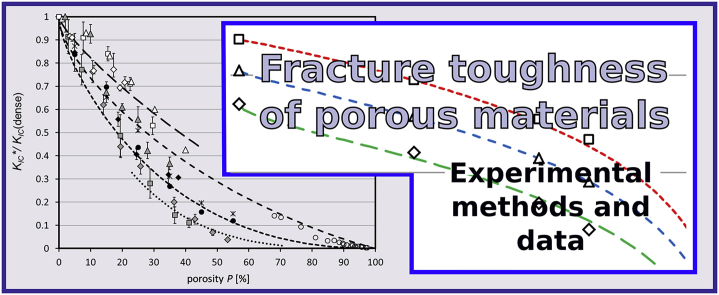

**Table:** Specifications table

Subject area	Physics, Materials Science
More specific subject area	Applied methods for measuring the fracture toughness of porous material
Type of data	Tables and figures
How data was acquired	Data taken from several published references or directly sent by the author
Data format	Analyzed
Experimental factors	Specimens were produced in different ways using standard procedures, like powder preparation, pressing, sintering under inert gas atmosphere, etc.
Experimental features	Different standard methods for the determination of the fracture toughness were applied, like SEVNB, SENB, SEPB, etc. (together with 3-point and 4-point bending), DCB, CT, etc.
Data source location	Data of nanoporous gold [2]: Helmholtz-Zentrum Geesthacht, Institut fuer Werkstoffforschung, Werkstoffmechanik, Max-Planck-Strasse 1, 21502 Geesthacht, Germany
Data accessibility	Data is with this article.
Related research article	H. Jelitto, G. A. Schneider, A geometric model for the fracture toughness of porous materials, Acta Mater. 151 (2018) 443–453 [1],

**Table:** 

**Value of the data** • We provide a compilation of mechanical data, especially of fracture toughness and Young's modulus, concerning porous materials from several research groups. • These experimental data allow for an easy check of theoretical models, including Ref. [1], [2], [3], which describes the fracture toughness of porous materials like advanced ceramics, polymers, and metals. • With the given experimental details of the measurements, the validity of the data can be estimated, particularly with regard to their application in Ref. [1], [2], [3].

## 1 Data

**Fig. 1:**
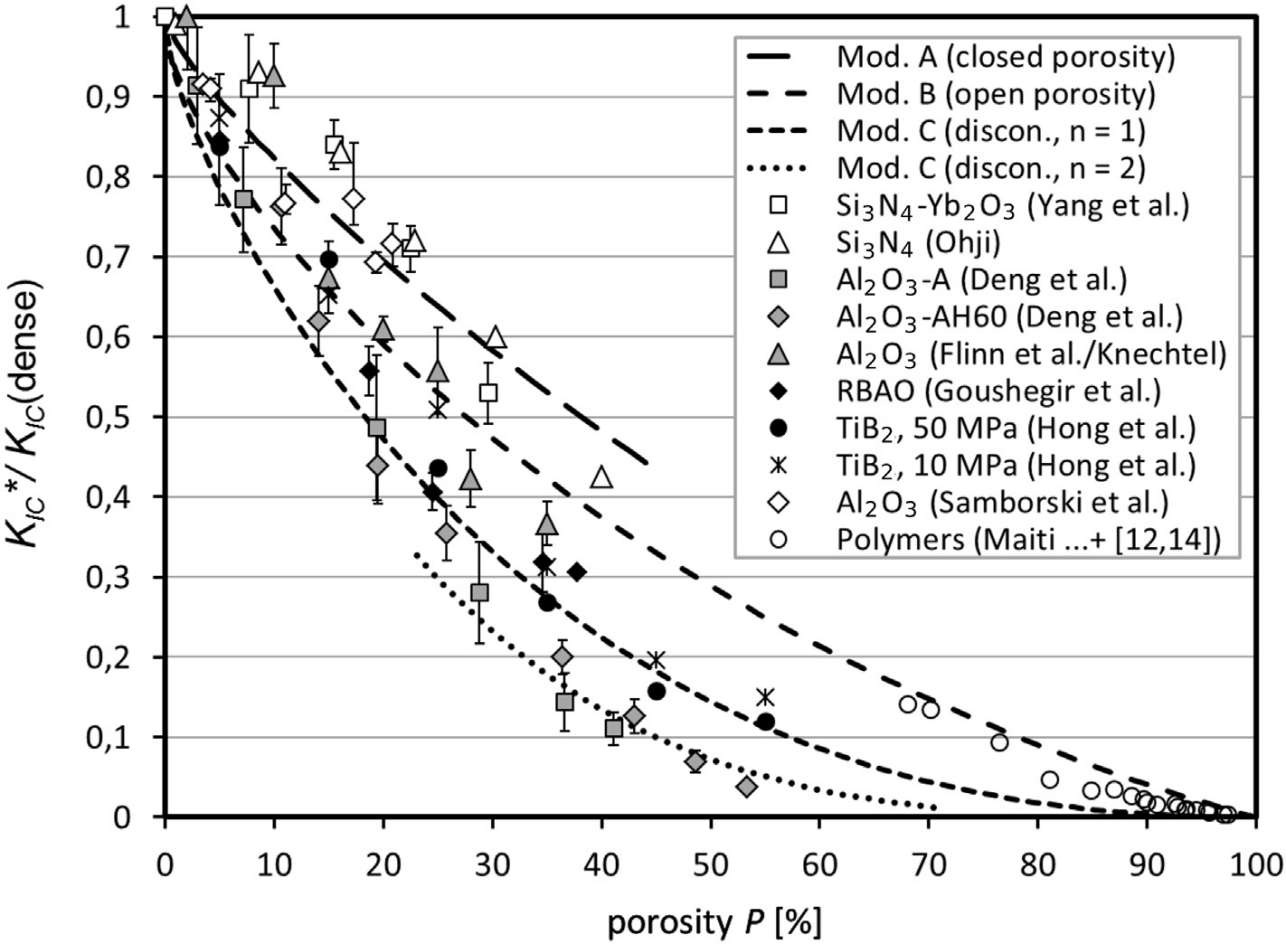
Overview of the measured normalized fracture toughness of different ceramic and polymer materials. As additional information, the main corresponding theoretical data from the model, described in Ref. [1], [2], [3], are provided.

**Fig. 2:**
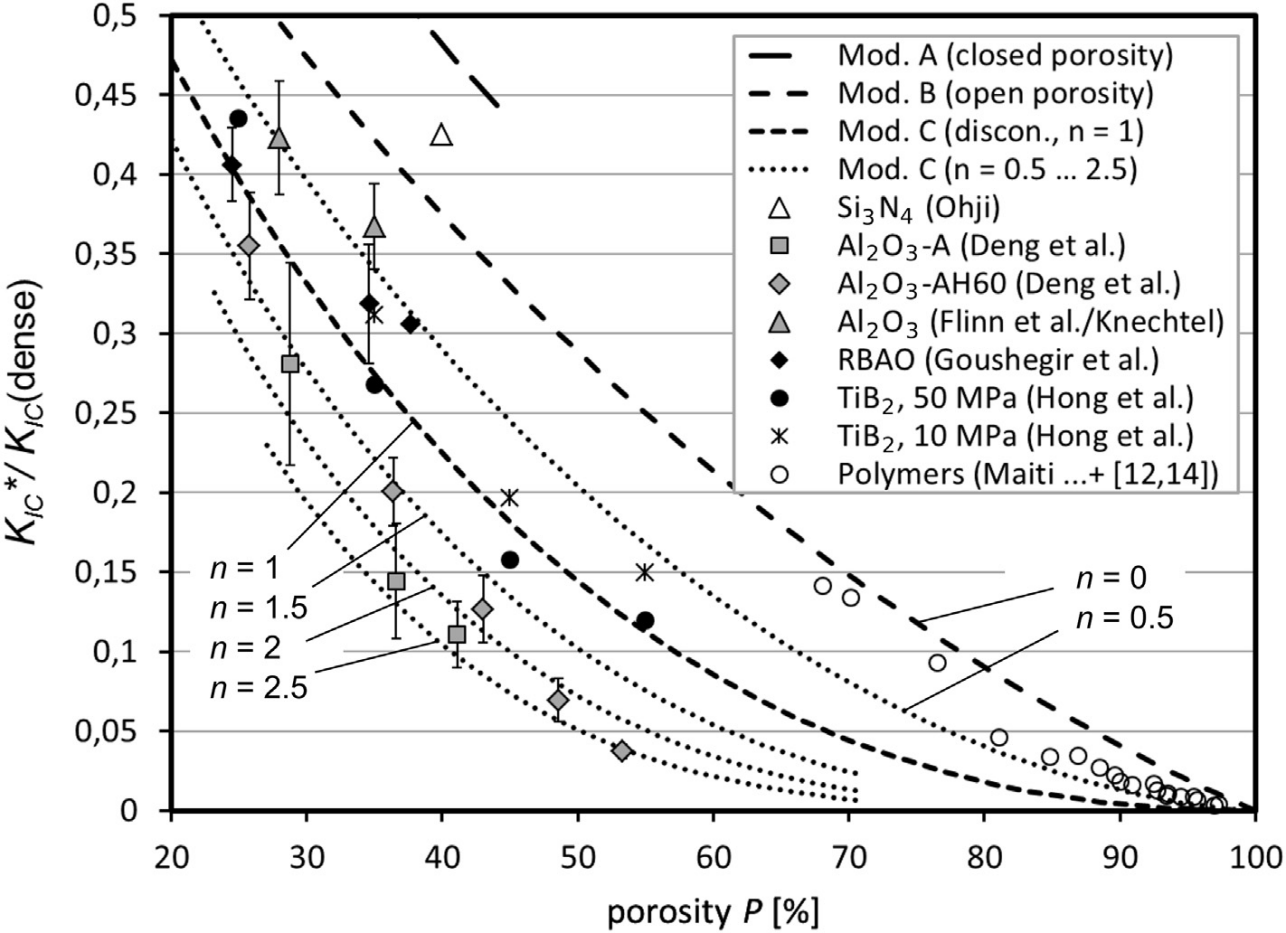
This figure shows a subarea of Fig. 1 together with additional model results. For more details, we refer to Ref. [1], [2], [3].

**Fig. 3:**
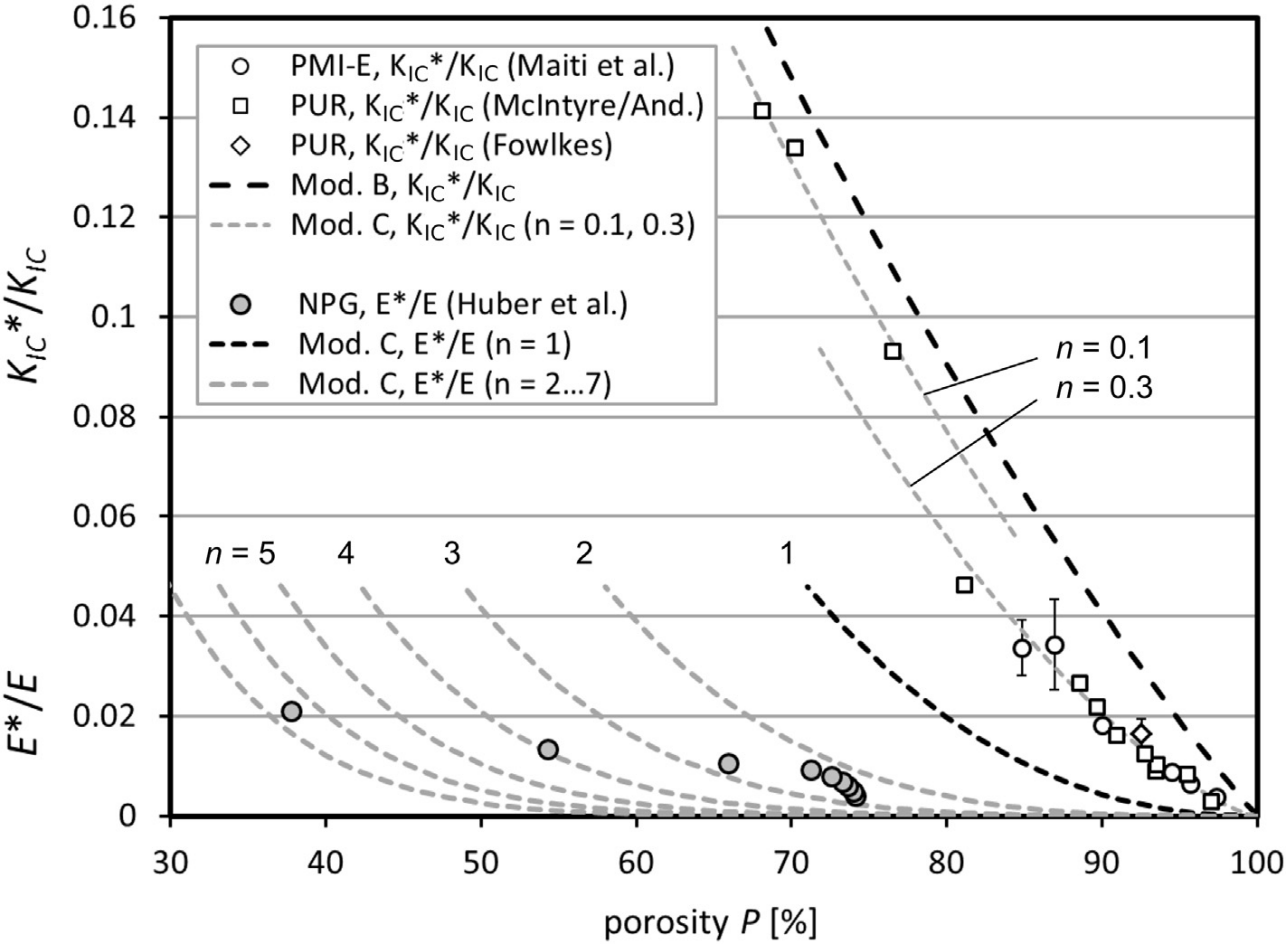
On the right side of the figure, the normalized fracture toughness of the tested polymer materials is shown [11], [12], [13], [14], which is again a scale magnification of the same data in Fig. 1, Fig. 2. Additionally, in the lower part of the diagram slightly left, we provide the normalized Young's moduli of nanoporous gold [2], together with results of model C, n = 1 … 7 [1], [2], [3]. The NPG was mechanically and successively loaded and unloaded.

**Fig. 4:**
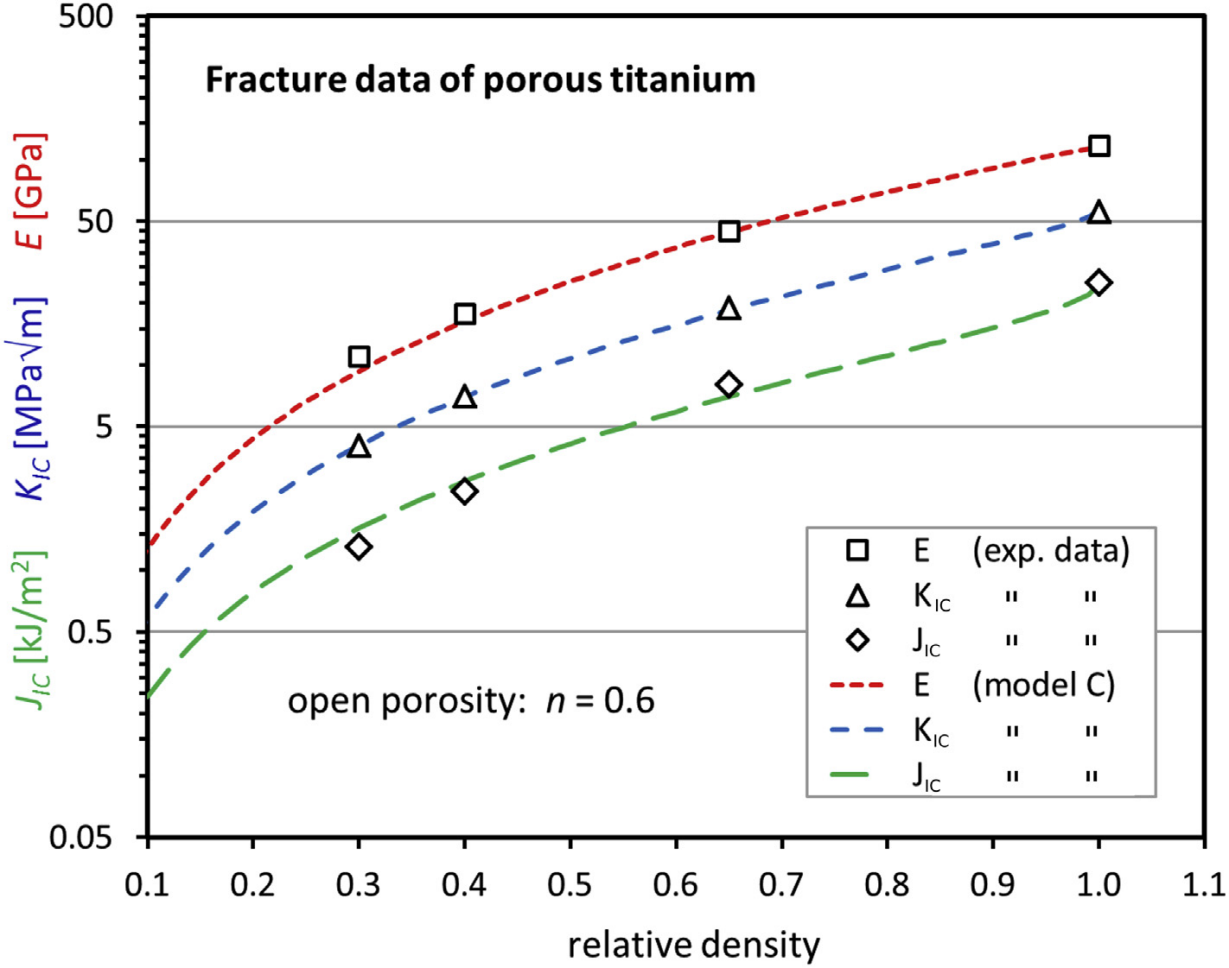
Young's modulus, fracture toughness, and initiation toughness of porous titanium as a function of the relative density. The presentation of the data in this figure is identical to Fig. 4 in the original reference [15] so that the figures can be compared easily. The theoretical curves are calculated with Eqs. (13), (16), and (21) of Ref. [1], [2], [3] and are multiplied by the measured values of the dense material.

**Fig. 5:**
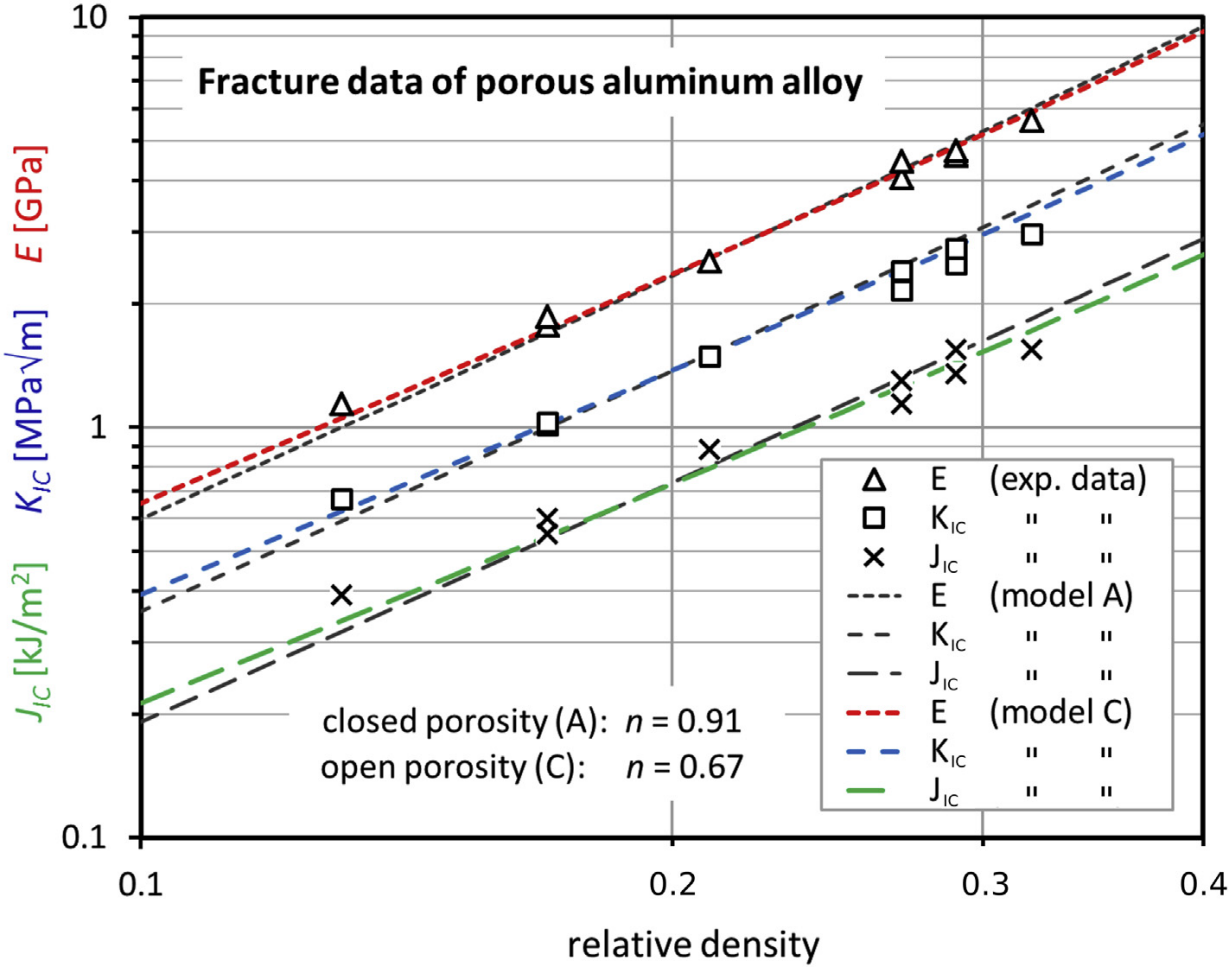
Young's modulus, fracture toughness, and initiation toughness of aluminum alloy (Al—Mg1—Si0.6) as a function of the relative density. As in Fig. 4, the presentation of the data is identical to that one in the original reference [17]. The theoretical colored curves (open porosity, model C) are calculated with Eqs. (13), (16), and (21) of Ref. [1]. The black dashed lines correspond to the extended model A (closed porosity) [21].

The main data are the normalized fracture toughness and corresponding errors (if available in the references) as a function of the normalized porosity. These data are carefully extracted graphically from figures in the given references [3], [4], [5], [6], [7], [8], [9], [10], [11], [12], [13], [14], [15], [16], [17]. Errors of a few percent like 1%–5% are possible, but they should be mostly below 2%. The only exception is Ref. [2], at which one of the authors sent us the numerical results. The graphical extraction is described in more detail in Ref. [1], [2], [3]. All of the data are provided in the Table 2, Table 3, Table 4, Table 5 and visualized in Fig. 1, Fig. 2, Fig. 3, Fig. 4, Fig. 5. After a major literature research and to the best of our knowledge, the given numerical data are not provided elsewhere. From the given references, the data of (mostly) isotropic materials and tests under quasi-static loading conditions are considered, in accordance to the model presented in Ref. [1], [2], [3]. For the sake of completeness, the data of the Young's moduli, used in Ref. [1], [2], [3], are provided in the Table 3, Table 4. The information and data comprise:

• Brief description of experimental design, materials, and methods
• Overview of the experimental information in Table 1
• Numerical experimental data in the Table 2, Table 3, Table 4, Table 5
• Visualization of the main experimental and theoretical data in Fig. 1, Fig. 2, Fig. 3, Fig. 4, Fig. 5

**Table 1:** Brief overview of the experimental methods concerning the fracture toughness of porous materials, measured by different research groups. The given “microstructural scale” is a rough estimate and corresponds to the average pore size (if not otherwise specified) or to the grain size. For more information, like the pore or grain size as a function of the porosity, see the respective references.

Reference	Material and its preparation	Experimental method	Microstructural scale [μm]
Yang et al. [3]	Si_3_N_4_, partial hot pressing (PHP)	SEPB, 3-point bending, average of 6 tests	0.1 … 0.5, diagram 4 in Ref. [3]
Ohji [4]	Si_3_N_4_, partial hot pressing (PHP)	Fracture energy by CNB (chevron-notched beam)	0.1 … 2, Figs. 1 and 3 in Ref. [4]
Deng et al. [5]	Al_2_O_3_ and Al_2_O_3_ + Al(OH)_3_, cold pressing, sintering in air	SENB, 3-point bending, pulse-echo method for Young's modulus	0.05 … 1,Figs. 6 and 9 in Ref. [5]
Flinn et al. [6] and Knechtel [7]	Al_2_O_3_, slip casting, different porosities by different sintering temperatures	SEPB (DIN 51109), relative crack length between 0.2 and 0.4	0.8 … 3 (grain size)
Goushegir et al. [8]	RBAO, uniaxial and cold isostatic pressing	SEVNB, 4-point bending, notch tip radius 10 μm	0.1 … 6 (grain size)
Hong et al. [9]	TiB_2_, uniaxial and cold isostatic pressing (10 and 50 MPa), sintering in vacuum	SENB, 3-point bending, relative notch depth 0.5, average of 3 tests	2 (particle size of powder)
Samborski and Sadowski [10]	Al_2_O_3_, (powder sintering technique)	SEVNB, 3-point bending, relative notch depth 0.175–0.325	–
Maiti et al. [11]	Foamed polymethacrylimid, (Rohacell, Rohm GmbH, Germany)	SEVNB, 3-point bending, relative notch depth 0.5	∼300 (for each porosity)
McIntyre et al. [12], Anderton [13]	Polyurethane foam, mixing of two commercial components	Single-edge-cracked specimen, tensile test	∼60 … 380 (mean cell size)
Fowlkes [14]	Polyurethane foam, center-cracked plate, double-edge-cracked plate, and single-edge-cracked tension specimen	Compliance method, DCB, and tensile tests with 3 sample geometries	average: 200 (∼150 … 400), estimated from Fig. 10a [14]
Huber et al. [2]	NPG, Master alloy Ag_75_Au_25_ melted, homogenized, and electro-chemically dealloyed	Successive compression, unloading in between (to determine Young's modulus)	∼0.1
Kashef et al. [15]	Titanium, particle size 45 μm, Ammonium bicarbonate as space holder, size 500–800 μm, mixed, pressed, heat treated, sintered	Compact tension method (ASTM E1820-08) [19], crack length by image processing and compliance method	500 … 800 (size of space holders)
McCullough et al. [17]	Aluminum alloy, Al—Mg1—Si0.6, specimens from sandwich panels of Alulight foam [17]	Compact tension method (ASTM E813-89) [20], crack extension by d.c. potential drop method, “initiation toughness”	∼500 … 1500 (depending on the relative density)

**Table 2:** Measured normalized fracture toughness and their errors as a function of the porosity. The data were graphically taken from the references. In four cases, only the absolute K_IC_-values were given. So, these data were normalized by dividing them by K_IC_(dense) of 5.44 [5], 4.16 [8], 3.21/4.18 [9], and 3.63 MPa√m [10], respectively. The latter numbers were obtained by extrapolation as described in Ref. [1], [2], [3]. The absolute errors, ΔK_IC_ (error bars), were also determined graphically from the references.

Reference (material)	Porosity [%]	K_IC_/K_IC_(dense)	ΔK_IC_/K_IC_(dense)
Yang et al. [3] (Si_3_N_4_)	0.0	1.0	0.051
	7.7	0.91	0.067
	15.5	0.84	0.031
	22.5	0.71	0.028
	29.6	0.53	0.038
Ohji [4] (Si_3_N_4_)	1.0	0.99	-
	8.64	0.93	-
	16.16	0.83	-
	22.9	0.72	-
	30.3	0.60	-
	40.0	0.425	-
Deng et al. [5] (Al_2_O_3_–A)	41.1	0.110	0.021
	36.6	0.144	0.036
	28.8	0.281	0.064
	19.4	0.487	0.091
	7.20	0.771	0.066
	2.93	0.913	0.073
Deng et al. [5] (Al_2_O_3_–AH60)	53.3	0.037	0.005
	48.6	0.069	0.014
	43.03	0.127	0.021
	36.4	0.200	0.021
	25.8	0.355	0.034
	19.5	0.439	0.048
	14.1	0.619	0.044
	10.67	0.763	0.048
Flinn et al. [6], Knechtel [7] (Al_2_O_3_)	35.0	0.367	0.027
	28.0	0.423	0.036
	25.0	0.558	0.054
	20.0	0.609	0.016
	15.0	0.674	0.045
	10.0	0.926	0.040
	2.0	1.00	0.066
S. M. Goushegir et al. [8] (RBAO)	37.7	0.306	-
	34.6	0.319	0.037
	24.5	0.406	0.023
	18.7	0.558	0.031
	5.0	0.846	0.082
Hong et al. [9] (TiB_2_, 50 MPa)	55.0	0.120	-
	45.0	0.158	-
	35.0	0.268	-
	25.0	0.435	-
	15.0	0.696	-
	5.0	0.837	-
Hong et al. [9] (TiB_2_, 10 MPa)	55.0	0.150	-
	45.0	0.196	-
	35.0	0.312	-
	25.0	0.508	-
	15.0	0.651	-
	5.0	0.872	-
Samborski et al. [10] (Al_2_O_3_)	3.50	0.915	+0.004/–0.010
	4.15	0.909	+0.014/–0.015
	11.05	0.766	+0.024/–0.012
	17.30	0.771	+0.071/–0.031
	19.35	0.693	+0.013/–0.014
	20.85	0.716	+0.024/–0.029
Maiti et al. [11] (polymethacrylimid)	97.34	0.0038	0.0008
	95.67	0.0064	0.0006
	94.47	0.0089	0.0009
	90.00	0.0183	0.0010
	86.95	0.0344	0.0091
	84.83	0.0337	0.0055
McIntyre et al. [12] (polyurethane)	96.99	0.0031	-
	95.45	0.0084	-
	93.47	0.0090	-
	93.55	0.0105	-
	92.75	0.0126	-
	90.93	0.0162	-
	89.65	0.0219	-
	88.54	0.0268	-
	81.12	0.0463	-
	76.51	0.0933	-
	70.16	0.1340	-
	68.05	0.1414	-
Fowlkes [14] (polyurethane)	92.54	0.0165	0.0030

**Table 3:** Measured Young's moduli as a function of the porosity. For a diagram of these numbers, see Fig. 10 in Ref. [1], [2], [3].

Reference (Material)	Relative density	E [MPa]
Deng et al. [5] (Al_2_O_3_–A)	0.5877	62.61
	0.6328	96.52
	0.7094	140.87
	0.8045	225.0
	0.9246	337.39
	0.9684	382.17
Deng et al. [5] (Al_2_O_3_–AH60)	0.4689	20.26
	0.5129	29.83
	0.5692	45.48
	0.6359	87.22
	0.7408	153.3
	0.8041	215.91
	0.8582	265.48
	0.8933	305.04
Deng et al. [5] (Al_2_O_3_–AH90)	0.3791	8.70
	0.4283	16.09
	0.4648	21.74
	0.543	50.65
Deng et al. [5] (Al_2_O_3_, ρ_0_ = 0.62)	0.7082	100.82
	0.7803	170.43
	0.85	230.43
	0.9594	363.36
Deng et al. [5] (Al_2_O_3_, ρ_0_ = 0.50)	0.5791	70.87
	0.65	120.87
	0.7291	185.65
	0.85	276.52

**Table 4:** Measured relative Young's modulus of nanoporous gold [2]. The first two columns were provided from one of the authors of the given reference. We normalized the data by dividing through the Young's modulus of the dense gold (81 GPa), specified in Ref. [2].

Reference (Material)	Porosity [%]	E [MPa]	E/E (dense)
Huber et al. [2] (NPG)	74.086	322.241	0.00398
	73.989	396.066	0.00489
	73.651	491.567	0.00607
	73.262	556.541	0.00687
	72.581	647.705	0.00800
	71.209	746.830	0.00922
	65.907	853.888	0.01054
	54.312	1096.29	0.01353
	37.738	1710.83	0.02112

**Table 5:** Measured Young's modulus, fracture toughness, and toughness of porous titanium [15] and porous aluminum alloy [17]. The quantities were extracted graphically from Fig. 4 in Ref. [15] and Fig. 10a) in Ref. [17]. The marked numbers at the bottom of the table are not provided in this reference: * taken from Ref. [22], ** by adapting the “ligament parameter” n of the model equations to the experimental data of Ref. [17], *** calculated from E and J_IC_ as before. In this case, the numbers of J_IC_ and K_IC_ vary slightly, depending on the kind of model (closed or open porosity).

Ref. (Material)	Rel. density	E [GPa]	J_IC_ [kJ/m^2^]	K_IC_ [MPa√m]
Kashef et al. [15] and Teoh et al. [16] (titanium)	0.30	11.0	1.3	4
	0.40	17.6	2.4	7
	0.65	44.7	8.0	18.95
	1.00	116	25	55.5
McCullough et al.[17] (Al-alloy)	0.13	1.14	0.39	0.668
	0.17	1.77	0.55	1.01
	0.17	1.86	0.60	1.03
	0.21	2.54	0.88	1.49
	0.27	4.05	1.14	2.16
	0.27	4.44	1.30	2.41
	0.29	4.60	1.35	2.50
	0.29	4.72	1.55	2.72
	0.32	5.59	1.55	2.96
(model C, open p.)	1	70*	26**	44.7***
(model A, closed p.)	1	70*	23**	42.1***

## 2 Experimental design, materials, and methods

In this section, we provide a brief description of the experimental details (if available) for each research group. This allows for a better understanding of the performed measurements, the results, and their reliability. The experimental methods, specified here, are mainly those, which are relevant for Ref. [1], [2], [3]. An abbreviated overview of the materials and methods concerning the fracture toughness measurements is given in Table 1. In each case, the sample preparation was more complicated than described here. Additional details are provided in the corresponding references. For an easier visualization, the results, shown in Fig 7 of Ref. [1], [2], [3], are displayed here in three Fig. 1, Fig. 2, Fig. 3 with different scales. This article covers a typical set of porous ceramics and polymers.

In order to complete the overview, results from porous metals are added. Although we checked about 20 publications concerning metallic foams, not much respective data of the toughness and fracture toughness exist. We found two papers in which the toughness (initiation toughness), the fracture toughness, and the Young's modulus of metallic foams are published. These three quantities are exactly those, which can be calculated by the model propounded in Ref. [1], [2], [3]. For detailed information concerning the results for ceramics and polymers, we refer to the latter reference.

### 2.1 Ceramics

***Yang et al.*** [3]: (Quotation) “Porous silicon nitride ceramic with a porosity from 0 to 0.3 was fabricated by partial hot pressing (PHP) of a powder mixture of α-Si_3_N_4_ and 5 wt% Yb_2_O_3_ as sintering additive. Irrespective of the porosity, the samples exhibited almost the same microstructural features including grain size, grain aspect ratio, and pore size” [3]. The aimed densities were achieved by the amount of starting powder in combination with a defined final mold volume during PHP (1800 °C). The sample dimensions were 3 × 4 × 42 mm^3^. The fracture toughness was determined by the single-edge-precracked beam method (SEPB, Japanese Industrial Standard R1607) in 3-point bending with a support distance of 16 mm and averaging from six tests. The loads for the Vickers indentations were 98 N or 196 N. This reference is the only one, concerning ceramics, where the amounts of open and closed porosity were determined separately. Below 8% the porosity was closed and above 15% mainly open – in between a mixture of open and closed porosity was found.

***Ohji*** [4]: Two types of silicon nitride were tested: isotropic and anisotropic porous Si_3_N_4_. In the latter case, the elongated grains were mainly aligned in one direction, but as already said, only tests of the isotropic material are considered here. Different porosities between 0% and 30% were obtained by using the PHP process. The porosity was controlled by the configuration of the carbon mold and the amount of powder. The other parameters were fixed, like powder mixture and sintering temperature of 1800 °C in nitrogen atmosphere for 2 hours. The fracture energy *γ*_*eff*_ was determined by the chevron-notched beam (CNB) test and the fracture toughness *K*_*IC*_ was converted from the relation: *γ*_*eff*_ = *K*_*IC*_^2^(1 – *ν*^2^)/(2*E*) with *ν* and *E* being the Poisson's ratio and the Young's modulus.

***Deng et al.*** [5]: Pure Al_2_O_3_ and a mixture of Al_2_O_3_ + Al(OH)_3_ were used as starting powder, having the designations A and AH. The relative amount of Al(OH)_3_ was 60% and 90%, indicated by AH60 and AH90, respectively. Green bodies were prepared by cold pressing and then sintered in air. Various porosities were obtained at different sintering temperatures from 1100 °C to 1450 °C for 30 minutes each. The specimens with dimensions of 3 × 4 × 40 mm^3^ were used for strength and toughness measurements. The fracture toughness was determined in the single-edge-notched beam (SENB) test with a notch depth of 2.0 mm and a notch width of 0.1 mm. Six measurements were done for each data point. The pulse-echo method, according to JIS R1602, yielded the Young's modulus of the porous alumina.

***Flinn et al.*** [6], ***Knechtel*** [7]: Beside other results, Flinn et al. published data from Knechtel, who tested alumina samples of different porosities [7]. The alumina (Alcoa CT 2000 SG) samples were prepared by slip casting. The mixture of 75 wt% Al_2_O_3_, 23.5 wt% H_2_O, 0.5 wt% steric stabilizer, and 1 wt% diluted soda solution resulted in a solid content of 45 vol%. After filling the slurry in plaster molds and drying, the green bodies got a relative density of 54%. During sintering, densities from 60 to 95% and grain sizes between 0.8 and 3 μm were obtained by temperatures between 1380 °C and 1650 °C (1 h). Sintering at 1680 °C for 4 hours yielded a density of 98%. The final bending bars had the dimensions 3 × 4 × 25 mm^3^. The fracture toughness was determined with the single-edge-precracked beam method (SEPB, DIN 51109), where the ratio of crack length to sample height was in the range 0.2–0.4.

***Goushegir et al.*** [8]: The preparation procedure of the RBAO precursor powder includes 40 vol% Al, 30 vol% fine grained Al_2_O_3_, 10 vol% coarse grained Al_2_O_3_, and 20 vol% fine grained TZ-3Y (yttria-stabilized zirconia). Additionally, alumina fibers were incorporated in order to get a full scale all-oxide composite with fiber volume fractions of 35–40%. Green bodies were obtained by uniaxial and subsequent cold isostatic pressing. However, for the measurement of the fracture toughness at different porosities, also monolithic (fiber-free) samples were prepared with dimensions of 3 × 4 × 40 mm^3^. They were tested by the single-edge V-notched beam (SEVNB) method in 4-point bending with a notch tip radius of 10 μm and support distances of 10 and 20 mm. The porosities were controlled by different sintering temperatures between 1100 °C and 1500 °C. The grain size increased with increasing sintering temperature.

***Hong et al.*** [9]: After appropriate preparation, TiB_2_ powder with a mean particle size of 2 μm was prepressed under uniaxial pressure and further compacted by (cold) isostatic pressing. Two different material variants were obtained by using 10 MPa and 50 MPa isostatic pressure. Afterwards, the samples were sintered under vacuum at temperatures from 1800 °C to 2000 °C. The fracture toughness was measured in 3-point bending with the singe-edge-notched bend (SENB) method. The beam dimensions were 2 × 4 × 22 mm^3^, the notch depth 2 mm, the notch tip radius 0.2 mm, and the support distance 16 mm. Each data point represents an average of three tested specimens.

***Samborski and Sadowski*** [10]: Porous ceramics of Al_2_O_3_ and MgO were prepared by powder sintering technique at the Institute of Electronic Materials Technology (Warsaw, Poland). The sintered bodies were cut to create specimens with a cross section of 3 × 4 mm^2^ and with a length of 40 mm (Al_2_O_3_) and 50 mm (MgO), respectively. Both types of materials were loaded quasi-statically and dynamically. For comparison with the model in Ref. [1], [2], [3], only the quasi-static tests of alumina were used. With the magnesia data, the extrapolation of *K*_*IC*_ to the density of the solid material, in order to normalize the fracture toughness, included too much uncertainty. The alumina tests were performed with the SEVNB method in 3-point bending with a support distance of 20 mm and a notch depth between 0.7 mm and 1.3 mm.

### 2.2 Polymers

***Maiti et al.*** [11]: Commercial foamed polymethacrylimid specimens (“Rohacell”, Rohm GmbH, Germany) were tested in 3-point bending (ASTM E-399-81) at room temperature. On the basis of the SEVNB method, the specimen dimensions were 25 × 50 × 250 mm^3^ with a relative notch depth of 0.5. The term “foamed” suggests that the material had closed porosity – at least partially – like many other polymer foams. This was not explicitly confirmed by the authors but, nevertheless, this seems obvious from their arguments on pages 213 and 215 in Ref. [11]. The pore size of approximately 300 μm was almost independent of the porosity. In their publication, Maiti et al. used also data from Refs. [12], [13], [14].

***McIntyre and Anderton*** [12]: (Quotation) “Rigid polyurethane foams were prepared from a commercial two component system, Propocon MR49 and Isocon M supplied by Lankro Chemicals Ltd.” Different porosities were obtained by different molds, containing the mixed raw material. The mold was open so that the material could expand freely, or it had a defined closed volume restricting the expansion to increase the density. The average pore size became larger with increasing porosity. The samples with dimensions of 5 × 35 × 150 mm^3^ were tested in tensile configuration (single-edge-cracked specimen) with a notch of different lengths up to 10 mm [13]. The amount of closed cells was determined with the ASTM Test-Method D1940-62T, which showed that the foams had predominantly closed porosity. (Remark: The used material was slightly anisotropic, as also in Ref. [14]. However, the data are well in the range of the data from Maiti et al. and so, this slight anisotropy was neglected by them [11] as also in Ref. [1], [2], [3].)

***Fowlkes*** [14]: Only one type of polyurethane foam with a relative porosity of approximately 92.5% was fabricated. Therefore, the toughness *G*_*Ic*_ was measured by using various methods: 1. compliance method, 2. double cantilever beam (DCB), 3. tensile tests with three different specimen geometries: a) center-cracked plate, b) double-edge-cracked plate, and c) single-edge-cracked tension specimen. The corresponding results agreed relatively well. The proportions of the specimens for the tensile tests correspond closely to the ASTM norm [18]. The relative density and the calculated normalized fracture toughness – applied in Ref. [1], [2], [3] – were taken from Ref. [11], where the respective result of Ref. [14] was used.

### 2.3 Metals

***Huber et al.*** [2]: Nanoporous gold was prepared by melting an Ag_75_Au_25_ alloy, homogenizing by vacuum annealing at 750 °C, cutting cylindrical samples with a wire saw after cooling, electro-chemical dealloying, and cleaning (rinsing) the sample with ultrapure water. The achieved porosity was 74 ± 1% and the mean ligament diameter 63 ± 6 nm. During the test, the sample was successively compressed and unloaded until the volume of the sample was reduced to half of its initial value. With these compression tests, only the Young's modulus was measured by evaluating the linear parts of the loading/unloading curves.

***Kashef et al.*** [15]: Porous titanium was produced by sintering of compacted mixtures of commercially available titanium powder (purity 99.9%, average particle size 45 μm) and space holder material (ammonium bicarbonate, NH_4_HCO_3_, particle size 500–800 μm). The achieved relative densities were 0.30 and 0.40. Green bodies were made by pressing with 200 MPa at room temperature. The space holders were removed by heat treatment at 100 °C for 10 hours and sintering took place at 1120 °C for 7 hours in a vacuum furnace. Then CT-specimens were wire cut to a size of 16 × 15.36 × 6.4 mm^3^ and fracture toughness testing was performed in accordance to ASTM E1820-08 [19]. The crack length was determined by the resistance curve procedure. From the *J*_*IC*_-curves, measured at stable crack growth, a conditional value of *J*_*IC*_ could be derived. The Young's modulus was determined by the elastic unloading compliance technique. As in Ref. [16], the fracture toughness was calculated by *K*_*IC*_ = √(*J*_*IC*_⋅*E′*) with *E’* = *E/*(1 –*ν*^2^) and *ν* = 0.3 being the Poisson ratio. Corresponding data for the relative density 0.65, used in Ref. [15], had been taken from Teoh et al. [16].

***McCullough et al.*** [17]: Closed cell aluminum-based foams (trade-name “Alulight”) with the composition AL-Mg1-Si0.6 and Al—Mg1—Si10 (wt%) were tested by compact tension for relative densities between 0.1 and 0.4. The CT-specimen geometry was 50 mm, measured from the center of the holes to the back side, and a thickness of 7.5 mm. The *J*-integral test procedure, according to ASTM E813-89 [20], allowed for the determination of the initiation toughness, *J*_*IC*_, being equal to the strain energy release rate (toughness), *G*_*C*_. The Young's modulus, *E*, was determined from the elastic unloading compliance as specified in the afore mentioned ASTM norm. Finally, *K*_*IC*_ could be calculated from *J*_*IC*_ and *E* according to the equations, already used by Kashef et al. (see above). Measurement of *J*_*IC*_ was performed using the single specimen technique. For the crack length, the DC potential drop method was applied and checked twice by compliance methods using back face clip gauge on the one hand and the displacement transducer on the other hand.

### 2.4 Additional information for the data visualization

For the calculation of the theoretical absolute values in Fig. 5 from the normalized curves, we need the quantities for the solid material. As these are not provided numerically in Ref. [17], we found the Young's modulus of 70 MPa for the material Al—Mg1—Si0.6 in Ref. [22]. The toughness J_IC_ was obtained by adapting the “ligament parameter” n in the model equations to the measured J_IC_-data. This is like a nonlinear extrapolation of the toughness to the dense material on the basis of the given model. Then, K_IC_ of the solid material was calculated as before.

For open porosity (model C), Eqs. (13), (16), and (21) of Ref. [1], [2], [3] are applied. For closed porosity, an extended version of model A [21] was used. This means that Eqs. (7), (14), and (19) of Ref. [1], [2], [3] are equipped with the additional factor (1–P)^n^, which is described in detail in Ref. [21]. Here, P represents the normalized porosity. For the calculation of the theoretical K_IC_ in Fig. 4, Fig. 5, E was replaced by E/(1–ν^2^) with ν = 0.3 in order to be compliant with the plane strain condition, assumed in Refs. [15], [17]. Note that for the equations of E, J_IC_, and K_IC_ (Fig. 4, Fig. 5) the same parameter, n, is used. The two model versions (open and closed porosity) in Fig. 5 yield similar data, but the “ligament parameters” n are different.
