# Supplementary material for: Fracture toughness of porous materials – Experimental methods and data
Source: Data Brief. 2019 Mar 7;23:103709. doi: 10.1016/j.dib.2019.103709 (PMC6660429; doi:10.1016/j.dib.2019.103709)
Supplement: Multimedia Component 1 [file mmc1.docx]

**Data article**

**Title:** Fracture Toughness of Porous Materials – Experimental Methods and Data

**Authors:** Hans Jelitto, Gerold A. Schneider

**Affiliations:** Hamburg University of Technology, Institute of Advanced Ceramics, Denickestrasse 15, 21073 Hamburg, Germany

**Contact email:**  [h.jelitto@tuhh.de](mailto:h.jelitto@tuhh.de)

**Declarations of interests:** none

**Keywords:** Fracture toughness, toughness, elastic properties, porosity, modeling
